# Haemoptysis in pregnancy caused by a well-differentiated fetal adenocarcinoma: a case report

**DOI:** 10.1186/1752-1947-4-17

**Published:** 2010-01-20

**Authors:** Rebecca J Thompson, Philip S Hasleton, Paul M Taylor, Mark Woodhead, Louise M Byrd

**Affiliations:** 1 Department of Obstetrics and Gynaecology, St Mary's Hospital, Hathersage Road, Manchester M13 9WL, UK; 2 Department of Histopathology, The University of Manchester, Oxford Road, Manchester M13 9PT, UK; 3 School of Medicine, The University of Manchester, Oxford Road, Manchester M13 9PT, UK; 4 Department of Radiology, Manchester Royal Infirmary, Oxford Road, Manchester M13 9WL, UK; 5 Department of Respiratory Medicine, Manchester Royal Infirmary, Oxford Road, Manchester M13 9WL, UK

## Abstract

**Introduction:**

Haemoptysis in pregnancy is frequently assumed to be caused by a pulmonary embolism. However, it can also be an indicator of serious pathology.

**Case presentation:**

We report the case of a 27-year-old Caucasian woman who presented with haemoptysis in pregnancy that was discovered to be caused by a well-differentiated fetal adenocarcinoma of the lung.

**Conclusion:**

This case demonstrates the importance of establishing an accurate diagnosis when a pregnant woman presents with haemoptysis and that more serious pathology should be considered if the clinical symptoms persist and/or the presumed diagnosis of pulmonary embolism is not confirmed.

## Introduction

When the symptom of haemoptysis occurs in pregnancy, it is frequently assumed to be caused by a pulmonary embolism, which is a relatively common pathology in pregnancy. However, as in the non-pregnant population, it can be caused by a number of other conditions and can be an indicator of serious pathology. Here we present a case of haemoptysis in pregnancy caused by a well-differentiated fetal adenocarcinoma of the lung.

## Case presentation

A 27-year-old Caucasian woman, gravida 2 para 1, booked at 13 weeks gestation. An ultrasound scan confirmed an estimated date of delivery of April 19, 2007. She was a fitness instructor, and had no significant past medical history, aside from a chest infection prior to this pregnancy that was treated with antibiotics by her General Practitioner. A smoker since the age of 17 years (approximately ten cigarettes per day), she had stopped six months previously.

The index pregnancy proceeded uneventfully until she presented with haemoptysis to her local hospital at 25 weeks gestation. A chest x-ray suggested a possible right-sided pneumonia. She was prescribed a course of antibiotics, and discharged home.

**Figure 1:**
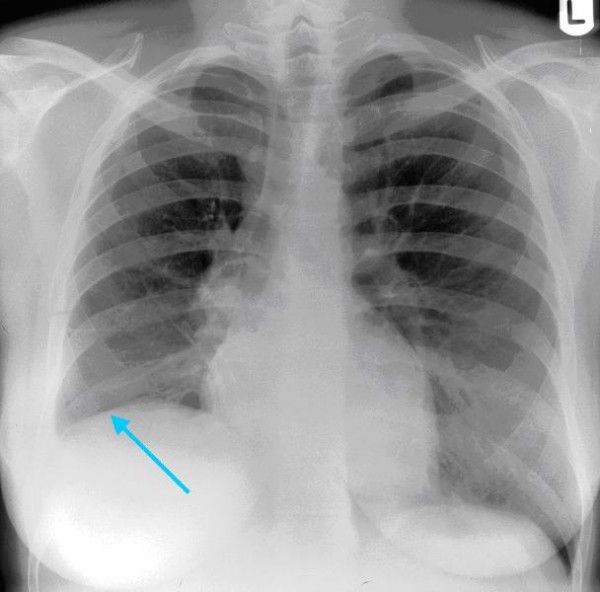
**Chest X-ray showing a small right basal pleural reaction and some right basal atelectasis**.

**Figure 2:**
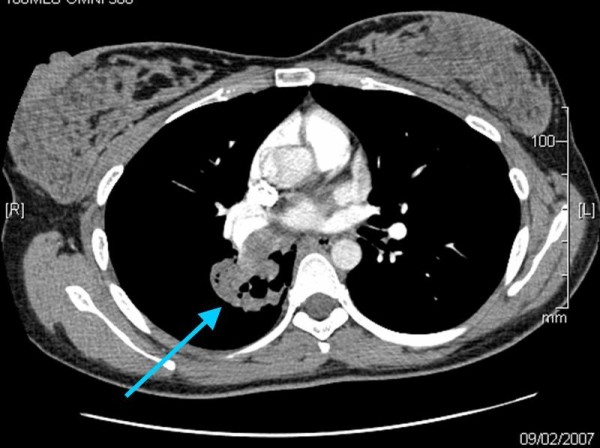
**Computed tomography pulmonary angiogram demonstrating cavitating mass in right lung**.

When seen in the antenatal clinic at 28 weeks gestation, she reported further episodes of haemoptysis. She was therefore admitted immediately for investigation. The working diagnosis was pulmonary embolism and as such she was fully anticoagulated with low molecular weight heparin (LMWH). Arterial blood gases breathing air were within normal limits (pO2 92.5 mmHg, 12.3 kPa; pCO2 33.6 mmHg 4.47 kPa), and sputum cultured normal upper respiratory tract flora. A chest x-ray (Figure 1) demonstrated a small right basal pleural reaction and some right basal atelectasis, which may indicate pulmonary embolic disease. A perfusion lung (V/Q) scan gave an indeterminate risk of pulmonary embolus. Nevertheless, despite several days of adequate treatment with LMWH (post dose Factor Xa 0.6), she had continued haemoptysis and a respiratory opinion was therefore sought. As a pulmonary embolus had as yet not been confirmed and the condition of the patient appeared to be worsening despite adequate therapy, bilateral leg Doppler's and a computed tomography pulmonary angiogram (CTPA) were performed. The former showed no evidence of a deep vein thrombosis. The latter revealed no pulmonary embolus but rather a cavitating mass of 4 cm in diameter within the right lung, immediately posterior to the hilum and occluding the lower lobe bronchus (Figure 2).

LMWH was discontinued and an urgent flexible bronchoscopy was performed 24 hours later. The view was obscured by active bleeding, and although a large tumour was seen in the right lower lobe bronchus it was not possible to undertake a biopsy. The woman was therefore discharged home and readmitted within the week for a scheduled rigid bronchoscopy. Biopsy was performed and unfortunately histopathology indicated a probable well-differentiated fetal adenocarcinoma.

Subsequent management was discussed in a Multidisciplinary Team Meeting, involving obstetrician, respiratory physician, cardiothoracic surgeon, radiologist and histopathologist. The possibility that there may be blastamous foci was raised. The need for appropriate staging, including abdominal computed tomography (CT) and positron emission tomography (PET) scan, with right pnuemonectomy should the tumour be localised, was agreed. As the safe use of PET in pregnancy has yet to be evaluated, it was decided that the baby should be delivered first.

Given that the woman was then 31+ 4 weeks gestation it was decided that she should receive corticosteroids over the following 48 hours (betamethasone 12 mg IM 24 hours apart), so as to encourage fetal lung maturation, with a view to delivery during 32 weeks of gestation. As there were some concerns as to whether Valsalva during second stage (that is active pushing) might precipitate significant haemoptysis from an apparently vascular tumour, an elective caesarean section was considered. However, following discussions with the woman, who was very keen to avoid such surgery, it was agreed that an induction should be attempted. Fortunately, following two does of prostaglandin, an amniotomy was possible. After that, she went on to deliver a male infant weighing 1970 gm.

Four days following delivery, a PET scan suggested that the tumour within the right bronchus was confined to the right lower and middle lobes of the lung.

Within ten days of delivery she was admitted for right lower and middle lobectomy. Several large sub-carinal glands were also removed. Surgery went well without event and the patient recovered rapidly and was discharged within a week.

Subsequent histology confirmed a well-differentiated fetal adenocarcinoma of the lung with one microscopic focus of blastoma, which appeared after sectioning the entire tumour. The mediastinal glands were not involved but tumour was present at the bronchial resection margin. As this tumour is not usually radiosensitive, it was decided that the remaining right upper lobe should be removed in order to affect a possible cure with the least possible chance of recurrence.

Again surgery was uneventful, and she made a quick recovery. However, histology from the completion pnemonectomy suggested the possibility of microscopic residual disease around the area of the stump of the right main bronchus. The clinical oncologists suggested treatment with radical external beam radiotherapy to try to prevent disease recurrence around the bronchial stump, which she underwent with minimal side effects.

A year following diagnosis, the patient is well and recurrence free. She does, however, report a persistent dry cough and breathlessness on severe exertion.

## Discussion

Well-differentiated fetal adenocarcinoma (WDFA) is classified by the World Health Organisation as a variant of adenocarcinoma. When fetal adenocarcinoma is associated with primitive blastemal stroma, it is classified as a pulmonary blastoma. In this case, there was only a microscopic focus of blastomatous tissue, so we were not totally certain that the categorisation into blastoma was correct. Pulmonary blastoma is a rare malignant tumour comprising 0.25 - 0.5% of all primary lung tumours [1-3]. Histologically, the WDFA element characteristically demonstrates neoplastic glands that resemble fetal lung and squamoid morules with clear nuclei [1]. The immature mesenchyme and epithelium mimic the embryonic lung at 10-16 weeks gestation [2]. The name WDFA is therefore derived from the histological appearances of the tumour. There is currently no known relationship between pulmonary blastoma and pregnancy. One study has hypothesised that oestrogen may be involved in the development of pulmonary blastoma through the oestrogen receptor-β [4], but in this case, the tumour was negative for this oestrogen receptor.

The peak incidence of pulmonary blastoma is 35-40 years of age, with an equal sex distribution. 80% of patients are smokers [3]. Our patient was somewhat younger, at only 27 years of age. Although she is currently a non-smoker, she had smoked for ten years prior to diagnosis. Patients may be asymptomatic or present with persistent cough, haemoptysis (as in this case) or chest pain.

The standard treatment for pulmonary blastoma is surgical resection [2,3], although there have been reports of limited success with adjuvant radiotherapy and chemotherapy [2]. The prognosis of pulmonary blastomas is poor, although compared with its biphasic counterpart, that of WDFA is better [5] (particularly when resection is complete), with a mortality rate of 14% and 52% respectively [3].

To the best of our knowledge this is the first reported case of WDFA diagnosed in pregnancy, although there has been a case of WDFA identified in a woman three months post partum [3], and one of a biphasic pulmonary blastoma diagnosed in pregnancy [6]. However, other types of lung carcinomas have been described in pregnancy. In most cases, the disease is reported as highly aggressive and frequently fatal.

## Conclusion

This case of well-differentiated fetal adenocarcinoma in pregnancy demonstrates the importance of establishing an accurate diagnosis when a pregnant woman presents with haemoptysis, cough and/or chest pain. When the clinical symptoms are persistent and/or a V/Q scan provides no logical explanation, it is important to seek advice from a senior respiratory physician, and consider undertaking a CTPA, both of which can play a vital role in identifying rare but life threatening lung pathology.

## Abbreviations

CTPA: computed tomography pulmonary angiogram; LMWH: low molecular weight heparin; PET: positron emission tomography; WDFA: well-differentiated fetal adenocarcinoma.

## Consent

Written informed consent was obtained from the patient for publication of this case report and accompanying images. A copy of the written consent is available for review by the journal's Editor-in-Chief.

## Competing interests

The authors declare that they have no competing interests.

## Authors' contributions

LMB, PSH, PMT and MW were involved in the multidisciplinary management of this case. RJT wrote the manuscript with the help and guidance of LMB. All authors reviewed the manuscript and contributed to the discussion.
